# The Autism Open Clinical Model (A.-O.C.M.) as a Phenomenological Framework for Prompt Design in Parent Training for Autism: Integrating Embodied Cognition and Artificial Intelligence

**DOI:** 10.3390/brainsci15111213

**Published:** 2025-11-11

**Authors:** Flavia Morfini, Sebastian G. D. Cesarano

**Affiliations:** SiPGI, Via Vittorio Veneto, 240, Torre Annunziata, 80058 Napoli, Italy; sebastian.cesarano84@gmail.com

**Keywords:** embodied mind, autism, parent training, artificial intelligence, A.-O.C.M., phenomenology, prompt design, ABA, clinical reasoning

## Abstract

*Background*/*Objectives*: In the treatment of autism spectrum disorders, families express the need for dedicated clinical spaces to manage emotional overload and to develop effective relational skills. Parent training addresses this need by supporting the parent–child relationship and fostering the child’s development. This study proposes a clinical protocol designed for psychotherapists and behavior analysts, based on the Autism Open Clinical Model (A.-O.C.M.), which integrates the rigor of Applied Behavior Analysis (ABA) with a phenomenological and embodied perspective. The model acknowledges technology—particularly artificial intelligence—as an opportunity to structure adaptive and personalized intervention tools. *Methods*: A multi-level prompt design system was developed, grounded in the principles of the A.-O.C.M. and integrated with generative AI. The tool employs clinical questions, semantic constraints, and levels of analysis to support the clinician’s reasoning and phenomenologically informed observation of behavior. *Results*: Recurrent relational patterns emerged in therapist–caregiver dynamics, allowing the identification of structural elements of the intersubjective field that are useful for personalizing interventions. In particular, prompt analysis highlighted how the quality of bodily and emotional attunement influences readiness for change, suggesting that intervention effectiveness increases when the clinician can adapt their style according to emerging phenomenological resonances. *Conclusions*: The design of clinical prompts rooted in embodied cognition and supported by AI represents a new frontier for psychotherapy that is more attuned to subjectivity. The A.-O.C.M. stands as a theoretical–clinical framework that integrates phenomenology and intelligent systems.

## 1. Introduction

The evolution of artificial intelligence is not an ideological choice; it is a concrete and irreversible condition of our time. One cannot simply be “for” or “against” technological progress: it exists, advances, and increasingly permeates the clinical field. It is therefore the therapist’s responsibility to take an informed and accountable stance, capable of orienting the use of technology toward ethical and relational purposes. The engineer designs algorithms and functional architectures, but does not define either the clinical objectives or the subjective contexts of application. It is the clinician’s task to design the interaction between humans and machines, training intelligent devices to respond to complex therapeutic goals rooted in the singularity of human experience.

From this perspective of clinical responsibility, the current state of the art in autism research highlights the need for models capable of integrating both the rehabilitative and the psychotherapeutic dimensions. International guidelines [1,2] confirm the importance of intensive, individualized, and early interventions, while at the same time emphasizing the centrality of family involvement and the work on relational dynamics. Recent studies have shown that autism cannot be understood exclusively as a neurocognitive deficit, but rather as a condition that entails specific modes of social learning [3] and intersubjective regulation [4]. Moreover, research in the field of embodied cognition has demonstrated how the body and perceptual–sensory processes play a decisive role in shaping subjective experience and developmental opportunities in autistic individuals [5]. In parallel, the international literature on the integration of artificial intelligence and psychology has shown significant advancements. Some studies have employed machine learning systems for language analysis and early detection of psychopathological symptoms [6], others have tested therapeutic chatbots capable of providing support in the field of mental health [7], while more recent research has applied neural networks to predict clinical outcomes and personalize psychological treatments [8].

However, at present, no clinical protocols have formalized a system of stratified prompts specifically applied to parent training for autism. It is precisely within this gap that the present work is situated, proposing itself as an innovative contribution while acknowledging the need for further developments and empirical validation, as will be highlighted throughout the article. Its aim is to present the Autism Open Clinical Model as a phenomenological and methodological framework for parent training in autism, from which an original protocol of stratified clinical prompt design originates. The work is addressed to professionals dealing with autism spectrum disorders with a specific professional profile: orientation in phenomenological psychotherapy and expertise in Applied Behavior Analysis, since the methodological complexity and the use of artificial intelligence tools require targeted competencies. This aspect will be further clarified in the Section 4. In this way, the reflection on the literature and on international scientific orientations does not serve as a mere background, but rather as a bridge toward the elaboration of the model presented here, showing how artificial intelligence makes it possible to structure adaptive and personalized protocols that do not replace clinical work but support it, offering new opportunities for observation and modulation of the intervention.

The aim of the present work is therefore twofold: on the one hand, to deepen and clarify the epistemological framework of the A.-O.C.M. as a scientifically oriented and phenomenologically grounded model; on the other, to show how such a framework finds concrete translation in the phased parent training protocol, supported by a system of stratified clinical prompts that follow a specific architectural and semantic logic. The Autism Open Clinical Model is first and foremost configured as a phenomenological model, whose guiding principle is Gestalt Therapy. The choice to ground the model in this matrix is not incidental: Gestalt Therapy provides the theoretical framework from which to derive the attention to the contact boundary, the valorization of creative adjustment, the conception of the organism as a body–mind unity, and responsibility in the present. On this phenomenological basis, the A.-O.C.M. consistently integrates principles derived from cognitive–behavioral psychotherapy and Applied Behavior Analysis (ABA), thereby combining clinical depth with operational rigor [9].

The epistemological structure of the model intertwines two distinct yet complementary sets of principles: on the one hand, philosophical principles, which orient the methodological vision; on the other, operational principles, which translate such foundations into concrete clinical choices. At the center lies the identification of active principles, not as the result of arbitrary selection, but of a rigorous review of the evidence-based scientific literature, integrated with the clinical experience of the authors. This methodological approach ensures that the variables assumed as central are indeed those with stronger empirical support, thereby reinforcing the theoretical solidity of the model [9,10].

The philosophical principles include scientific doubt, understood as constant critical inquiry that keeps the connection between clinical work and research alive; the principle of experimentation, which requires objective observation, accurate description, and operationalization of concepts; and the criterion of parsimony, which calls for privileging simple explanations before more complex hypotheses. The operational principles extend this framework into clinical work: the recognition of the right to diversity, creative adjustment with attention to the contact boundary, coherence between actions and objectives, centrality of the present, and personal responsibility. The organism is considered as a body–mind unity, self-regulated and oriented toward realization, consistent with a phenomenological approach that attributes meaning to observed behaviors without reducing them to mentalistic explanations [9].

From this perspective, clinical practice is conceived as a craftwork sustained by method: each therapeutic intervention is unique, tailored to the specificity of the individual and the context, the product of creativity and clinical intuition, but always accompanied by empirical verification and the operationalization of variables, thereby ensuring rigor and replicability [9,10]. Integration is understood as a dynamic process of bidirectional translation, in which complex concepts of an intrapsychic and relational nature are translated into observable and measurable categories, while behaviors are reconnected to clinical and phenomenological meanings. It is within this tension between rigor and openness, between operationalization and phenomenology, that the methodological innovation of the A.-O.C.M. resides, positioning it as a solid theoretical framework still in the process of empirical consolidation.

Within this framework, the structure of the present article can also be better understood: the integrative logic of the model and its theoretical depth find expression in the development of the protocol, articulated into phases and transformative nodes. The protocol translates its founding principles into operational practice through the description of a stratified prompt scheme. To make the exposition more concrete, the implementation of fragments of clinical cases is presented.

This results in a framework that combines flexibility, creativity, and methodological rigor, the distinctive hallmark of the A.-O.C.M. The very designation of an “open model” underscores this vocation: an experimental model is destined to remain such, as it is designed to be enriched by the contributions of other scholars and to incorporate new evidence. Openness is not merely a methodological attitude but also an epistemological choice, implying readiness for significant evolutions both on the theoretical and on the technological levels, including the development of clinical tools anchored in the paradigm of embodied mind. It should be noted that the anchoring to the paradigm of embodied mind represents a recent evolution of the A.-O.C.M. and is the subject of a forthcoming scientific publication. In this perspective, the body is assumed as the primary site of clinical meaning, intersubjective resonance, and regulation, with direct implications for observation, data collection, and intervention modulation. For this reason, the model has declared from its inception an openness to contributions from scholars of different orientations, in a perspective of shared growth and progressive theoretical and applicative refinement. Along this line of development, the A.-O.C.M. also demonstrates its attentiveness to contemporary evolutions and to the needs of the clinical population with autism: hence, the choice to consider engagement with artificial intelligence as essential, as evidenced by the preprint on AIDA (Artificial Intelligence, Domotics, and Applied Behavior Analysis), currently under review [11]. Although the model was formally established in 2023, its genesis dates back to the article “Criteria for an Integrated Clinical Model” [10], which outlined its original idea. In the following years, this reasoning was progressively enriched through several scientific contributions that, although not yet naming it explicitly, referred to this integrated clinical model until its more complete definition as the Autism Open Clinical Model. In this evolution, subsequent articles developed reflections and applications that can be traced back to its conceptual framework [10,11].

The construction and development of the A.-O.C.M. is therefore part of a pathway of gradual growth, supported by the twenty years of clinical experience of the authors. From daily clinical practice arose the project of progressively turning the model into an evidence-based approach: an objective pursued through a gradual process of theoretical reflections, publications, and empirical verifications [12]. In this sense, the A.-O.C.M. seeks to provide a complex and articulated response to a phenomenon that is itself complex and articulated, such as autism. The difficulty lies not only in the clinical picture but also in the tendency of the social system to provide “autistic” responses, rigidified into omnipotent and self-referential structures in which each subsystem tends to perceive itself as self-sufficient. The A.-O.C.M., by contrast, is founded on the principle of networking and teamwork, which do not remain abstract concepts but find concrete translation in daily clinical practice. Within this framework, contact with the network itself becomes a clinical action, embedded within a rigorously applied protocol, thereby making operative those principles of collaboration and integration that too often remain merely declared.

This perspective highlights the methodological foundation on which the clinical protocol for parent training in autism is built: a defined theoretical framework that finds its operational translation in a phased protocol, capable of guiding clinical work and systematizing the application of the identified principles [13].

## 2. Materials and Methods

As outlined above, the present manuscript represents a step in a complex and gradual research process that has traced the scientific trajectory leading to this work. The theoretical–methodological framework described thus leads to the structuring of an operational parent training protocol, understood not as a prescriptive and rigid sequence of actions, but as a flexible framework capable of adapting to the specific characteristics of the family and of accompanying caregivers in a pathway of awareness, reorganization, and change in relation to their child’s diagnosis. The complexity of the protocol, with its structuring into phases and their associated nodes, allows for an in-depth analysis of the numerous variables that characterize the family processes of individuals with autism spectrum disorder.

Within this protocol, the following aspects play a central role:

Needs analysis: a systematic process aimed at identifying the family’s needs, difficulties, and resources, in order to define realistic and clinically relevant objectives.

Definition of baselines: identification and initial measurement of target behaviors or indicators of family functioning, serving as a reference point for evaluating the effectiveness of interventions over time.

Shared responsibility of caregivers: active and joint involvement of parents and/or reference figures, conceived not as mere recipients but as co-protagonists of the therapeutic process.

Integration of observational data into applicable clinical tools: structured use of behavioral observations collected in natural contexts, translated into operational tools (grids, forms, protocols) that guide clinical decision-making and allow progress monitoring.

### 2.1. Phases of the Protocol

#### 2.1.1. Phase 1—Welcoming and Exploring the Family Context

The first phase is dedicated to building the therapeutic contract, integrating needs analysis with the exploration of the request for help. This is the stage where the foundations of an alliance are laid, recognizing the assisted individual as an active subject in their own path, and the caregivers as clinical partners. The work takes shape through an exploratory dialogue that does not force but accompanies, welcoming resistances as part of a subjectively functional temporary balance. Intense emotions often emerge from the confrontation between the “imagined child” and the “encountered child”: grief, anger, guilt, shame, or loneliness. Allowing space for these experiences opens a gateway to authenticity within the clinical relationship. At the same time, the impact of the diagnosis on family organization, the parental burden, the couple’s relationship, and support networks is explored, thus making visible the complexity of the system in which the child is embedded.

#### 2.1.2. Phase 2—Redefining Parental Identity and Expectations

This phase is dedicated to sharing clear and updated information on the child’s neuropsychiatric and behavioral functioning [14]. Parents are guided to recognize problem behaviors within a meaningful framework, learning systematic observation methods that allow them to identify the conditions of onset and maintenance. Understanding the logic underlying the behavior reduces the sense of unpredictability, strengthens reflective capacity, and fosters a calmer and more aware reading of everyday difficulties. The goal is not only to provide knowledge but to transform it into useful tools for orienting perception and promoting a more authentic contact with the child’s experience.

#### 2.1.3. Phase 3—Supporting the Emotional Bond and Secure Attachment

Based on the information collected and the shared objectives, clinical prescriptions are provided and formalized in clear and detailed protocols. Each indication specifies what to observe and how to intervene, defining the frequency, duration, intensity, and form of the expected behaviors. The proposed strategies aim to reduce dysfunctional behaviors and to promote adaptive skills, with constant attention to concreteness and operational clarity. It is in this phase that the theoretical framework is translated into targeted actions: parents receive practical tools, immediately applicable, which become an integral part of daily life.

#### 2.1.4. Phase 4—Integration of the Body, Empathy, and Embodied Communication

Parents are engaged in a process of direct experimentation with the strategies learned. Lived experience becomes an opportunity for transformation: theoretical understanding is translated into action, action becomes an occasion for contact, and contact opens the way to new forms of awareness. Family dynamics are observed and re-elaborated, fostering greater openness to the relationship with the child and with the entire family system. This is a moment in which theory and practice intertwine, and learning becomes embodied within the relationship itself.

#### 2.1.5. Phase 5—Symbolic Reorganization and Shared Narration

At this stage of the process, feedback from parents is collected, as they express their perception of the effectiveness of the tools learned and of the quality of the therapeutic relationship. Their narratives are integrated with the observational data gathered by the clinician and with quantitative indicators, thus constructing a comprehensive picture of the progress of the treatment. Evaluation is not only measurement: it is also a restitution of meaning, achieved through the convergence of what parents experience, the observable data, and the predefined objectives. This interplay between different dimensions adds depth and reliability to the results [15].

#### 2.1.6. Phase 6—Monitoring and Generalization

The final phase is oriented toward consolidating progress and extending it across the various life contexts of the child and the family. The goal is to foster strategic autonomy in parents, enabling them to adapt interventions to daily situations and to the child’s developmental trajectory. Generalization does not concern only the application of techniques but also the consolidation of a new attitude: the ability to modulate one’s responses, to read the child’s signals, and to transform difficulties into opportunities for shared growth. It is here that the process opens to the future, ensuring continuity of the work accomplished and anchoring it in the real life of the family [16].

**Figure 1:**
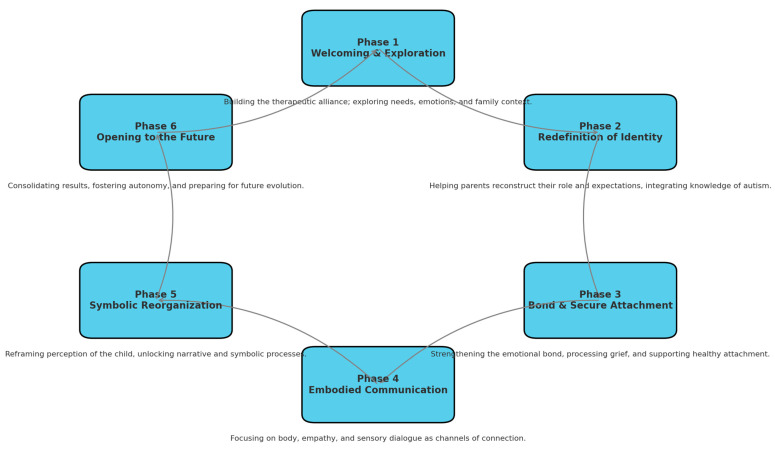
Six phases of the A.-O.C.M. Parent Training Protocol.

For clarity, Figure 1 provides a schematic representation of the six phases of the protocol.

The diagram below illustrates the six interconnected phases of the A.-O.C.M. Parent Training Protocol. Each phase is fluid, non-linear, and may re-emerge depending on the clinical context.

### 2.2. Phases and Transformative Nodes

The six phases of the clinical protocol are articulated in light of the phenomenological clinical dimensions and the 24 transformative nodes of the A.-O.C.M. Parent Training. Each phase includes and activates specific nodes, selected according to their impact on the evolutionary dynamics of the family system and on the transformation of the parental experience.

#### 2.2.1. Phase 1—Welcoming and Exploring the Family Context

##### Node 1—The Non-Integrated Parental Self

The parent oscillates between contrasting self-images: omnipotent and failed, present and distant. The transformative node lies in recognizing these dissociated parts and integrating a more authentic and imperfect self-image.

##### Node 5—The Denied Relational Need

Some parents convince themselves they do not need support or affection, shielding themselves from painful attachment. The transformative node enables the recognition of the legitimacy and value of one’s own need.

##### Node 6—Failure as Identity

Every difficulty of the child is internalized as proof of being a “wrong” parent. This node invites disidentification from guilt as an identity core.

##### Node 13—Who Am I Now?

Following the diagnosis, the definition of oneself as mother or father changes radically. This is an existential passage in which one’s personal history is reformulated. The transformative node lies in the possibility of redefining one’s own value and meaning.

#### 2.2.2. Phase 2—Redefining Parental Identity and Expectations

##### Node 2—Control as a Defense Against Chaos

The need to rigidly plan everything serves as protection against anxiety. The transformative node lies in learning to tolerate complexity and co-create with uncertainty.

##### Node 11—The Imagined Child that Overshadows the Encountered Child

Attachment to an imagined child obscures the child encountered in the here and now. The node enables parents to let go of the ideal and to embrace the real encounter.

##### Node 16—The Desire for Conformity

The parent may experience a painful desire for conformity, perceiving the family as “abnormal”. Transformation occurs through embracing difference as part of identity, not as a deviation to be corrected.

##### Node 18—The Punitive Index

The need to assign blame—to the partner, the child, or oneself—is a defensive strategy to control emotional chaos. Transformation emerges when blame is abandoned in favor of shared responsibility and meaning.

#### 2.2.3. Phase 3—Supporting the Emotional Bond and Secure Attachment

##### Node 7—Reactive Attachment

The bond with the child becomes ambivalent: oscillating between over-involvement and rejection. The node lies in building a synchronous, non-reactive attachment.

##### Node 10—The Child as an Evoker of Unprocessed Pain

The child may reactivate, in the parents’ perception, painful experiences or traumatic events that have not yet been integrated. Transformation consists of recognizing and giving form to this original pain, separating it from the current relationship with the child.

##### Node 12—The Child as a Space of Projection for Parental Conflicts

In some situations, the autistic child may be involved, even unconsciously, in the unresolved tensions of the parental couple. The transformative node enables the child to be freed from triangulating roles, restoring a more authentic and protected relational space.

##### Node 17—Rejection of Need

Manifested as affective avoidance, the parent deprives themselves of authentic contact. Working on this node means reintegrating vulnerability as a resource, not as a threat.

#### 2.2.4. Phase 4—Integration of the Body, Empathy, and Embodied Communication

##### Node 4—Disembodied Empathy

The parent “understands everything” but does not feel. Contact is purely cognitive. This node invites reintegrating the body into empathy, returning to feeling the child through all channels.

##### Node 8—The Disconnected Parental Body

The parent is absent from their own body. The node reactivates embodied presence.

##### Node 19—The Body as a Foreign Place

Touching and nurturing become technical gestures. The node restores the body’s affective function.

##### Node 20—The Uncoordinated Rhythm

The parent does not enter the child’s time. The node concerns rhythmic co-regulation.

##### Node 21—The Blocked Gesture

The body is restrained. The node releases bodily expressiveness.

##### Node 22—The Traumatic Touch

Physical contact is experienced as invasive. The node redefines touch as care.

##### Node 23—The Child’s Dissociated Embodiment

The child is perceived as a function. The node restores dignity and vitality to their body.

##### Node 24—The Interrupted Sensory Dialogue

Sight, touch, and sound are no longer communicative channels. The node reactivates embodied communication.

#### 2.2.5. Phase 5—Symbolic Reorganization and Shared Narration

##### Node 3—Fragmented Perception of the Child

The child is seen in parts. The node consists of reintegrating a global and affective vision of the child.

##### Node 9—Block of Symbolic Generalization

Meanings remain frozen. The node unlocks symbolic fluidity and makes learning embodied.

##### Node 14—The Blocked Narrative of the Relationship

When the parent cannot narrate the relationship story with the child, trauma is often present. The node reactivates narration as care.

##### Node 15—Repressed Impulsivity

Anger and fear are inhibited. The node helps access and modulate emotion without outbursts or suppression.

#### 2.2.6. Phase 6—Monitoring and Generalization

This phase embraces and synthesizes the processes activated in the previous phases, preparing for closure and the next evolutionary step.

### 2.3. Clinical Fragments—Case 1 Family A

#### 2.3.1. Phase 1

Two brothers, both diagnosed with level 1 autism, without cognitive disabilities or language impairment. The parents’ initial request was generic and poorly structured. Phenomenological observation revealed poorly functional family boundaries, a normative climate, emotional inhibition, and an implicit fear of disapproval.

The analysis of the request made it possible to clarify the parents’ implicit expectations, highlighting a sense of disorientation that oscillated between the desire to delegate change to the child and the fear of directly confronting their own vulnerabilities.

A baseline was defined through the topographical description of problem behaviors (PBs), observed as very frequent and pervasive. To ensure systematic monitoring, parents were provided with data collection forms to be completed over 7 consecutive days, in which behaviors were recorded based on frequency.

These tools clarified the distinction between the actual problem behavior and that perceived as such, fostering an initial step toward the shared redefinition of difficulties.Indicators consistent with the following:

- Node 1 (Non-integrated parental self)—in the difficulty of maintaining continuity in the parental role, with oscillations between involvement and withdrawal.
- Node 5 (Denied relational need)—in the tendency not to openly express the need for emotional support and to minimize fatigue.
- Node 6 (Failure as identity)—in associating the child’s difficulties with a self-critical perception of oneself as a parent.
- Node 13 (Who am I now?)—in the sense of identity disorientation and in the fragility of the parental role following the diagnosis.

#### 2.3.2. Phase 2

The parents maintained a defensive stance, delegating change to the children. The therapeutic contract was built on the basis of this analysis, defining specific areas of intervention and transforming observational data into clear objectives. For each objective, procedures, application techniques, and expected timelines were identified, in order to ensure traceability and transparency of the therapeutic process. The therapeutic contract was oriented toward redefining family boundaries, with an initial parent training intervention (12 sessions) and individual psychotherapy for the child with greater relational difficulties.

Indicators are consistent with the following:

- Node 2 (Control as defense against chaos)—in the difficulty tolerating uncertainty and the need to contain change.
- Node 11 (The imagined child that nullifies the encountered child)

Attachment to an imagined child overshadows the child encountered in the here and now. This node enables parents to let go of the ideal and to embrace the true encounter.

- Node 16 (Desire for conformity)—underlying rigid expectations.
- Node 18 (Punitive index)—in the tendency to place responsibility for change on the children.

#### 2.3.3. Phase 3

The work focused on raising awareness of implicit punitive dynamics, children’s separation anxiety, and parental confusion between actual problem behaviors and those merely perceived as such.

The implementation of data collection forms and educational strategies enabled parents to more clearly recognize the function of behaviors, progressively reducing emotional reactivity and increasing educational consistency. Outcomes showed a significant reduction in problem behaviors, shifting from very frequent and pervasive to isolated episodes, thereby strengthening parental functioning and enhancing tolerance of emotional contact.

Indicators are consistent with the following:

- Node 7 (Reactive attachment)—in the alternation between moments of affection and signals of implicit threat.
- Node 12 (Child as a space interposed between parents)—in the presence of indirect triangular dynamics related to rules.
- Node 17 (Rejection of need)—in the difficulty of recognizing and sharing one’s parental vulnerability.

#### 2.3.4. Phase 4

Micro-observations and in vivo experiments to transform communicative automatisms and support the children’s emotional expression without inhibitory reactions.

Clinical observations showed that working on the body and embodied communication enabled parents to modulate their interactive rhythm, reducing rigidity and fostering more authentic moments of emotional attunement.

Indicators are consistent with the following:

- Node 4 (Disembodied empathy)—tendency to manage interactions cognitively rather than affectively.
- Node 20 (Out-of-sync rhythm)—difficulties in co-regulating during interactions.
- Node 21 (Blocked gesture)—in restraining bodily expressiveness.
- Node 24 (Interrupted sensory dialogue)—in the limited openness to non-verbal channels.

#### 2.3.5. Phase 5

Greater tolerance for not knowing, reduction in directive verbalization, and differentiation between the parents’ own experiences and those of the children.

The treatment plan included moments of narrative re-elaboration, in which parents were guided to distinguish between personal projections and actual observed behaviors, fostering greater symbolic integration.

Indicators are consistent with the following:

- Node 3 (Fragmented perception of the child)—initially present and progressively reduced.
- Node 14 (Blocked narrative of the relationship)—progressively unlocked through shared feedback.
- Node 15 (Repressed impulsivity)—in the more balanced management of emotional reactions [14].

#### 2.3.6. Phase 6

Consolidation of permeable communicative spaces, relational reading of behavior, and increased capacity for educational modulation.

The therapeutic contract was revisited in the final phase to provide parents with feedback on the path undertaken: the initial baseline, characterized by frequent and pervasive problem behaviors, was transformed into an outcome with problem behaviors reduced to isolated episodes, integrated within a new parental capacity to regulate contact and emotional containment.

This phase synthesizes and integrates the processes activated in the previous nodes.

### 2.4. Clinical Fragments—Case 2—Family B

#### 2.4.1. Phase 1

An 11-year-old child with level 2 autism. The child’s identity was reinforced as a “naughty child” by both the family and school environment. Initial parental resistance to parent training was linked to emotional difficulties in accessing the pain associated with the diagnosis.

In this phase, the analysis of the request was carried out, highlighting the caregivers’ implicit need to reduce problem behaviors and to improve the child’s emotional regulation. At the same time, the therapeutic contract was defined, establishing shared objectives and timelines for evaluation.

Indicators are consistent with the following:

- Node 11 (Ideal image that erases the real child)—reflected in the difficulty in fully accepting the real child.
- Node 16 (Desire for conformity)—evident in the constant comparison with an implicit model of typical development.

#### 2.4.2. Phase 2

The identification of problem behaviors maintained by relational control was carried out. The baseline highlighted behaviors topographically described as prolonged yelling and protesting in response to requests, attempts at manipulation through strategic crying, verbal aggression such as insults, and frequent interruptions of activities. These behaviors were observed as very frequent and pervasive in family and school life. Parents were trained in ABA procedures of extinction and differential reinforcement [14], with initial resistance overcome through motivational engagement. Data collection was conducted using forms provided to caregivers, to be completed over 7 consecutive days, recording the frequency of behaviors.

Indicators are consistent with the following:

- Node 2 (Control as defense against chaos)—shown in the rigid management of behaviors and difficulty tolerating the unexpected.
- Node 18 (Punitive index)—tendency to interpret the child’s behavior in terms of blame or provocation.

#### 2.4.3. Phase 3

A reduction in manipulative behaviors and reinforcement of alternative behaviors was observed, with specific attention to the child’s empathic potential. The area of intervention focused on behavioral regulation, the use of appropriate verbal requests, and the modulation of frustration. In the treatment plan, specific procedures, reinforcement techniques, and expected timelines for evaluation were identified for each objective.

Indicators are consistent with the following:

- Node 7 (Reactive attachment)—implicit in alternating behaviors of seeking contact and manipulative control.
- Node 17 (Rejection of need)—observed when the child does not verbalize needs directly, instead expressing them indirectly.

#### 2.4.4. Phase 4

The work focused on managing emotional dysregulation during sessions, promptly interrupting dysfunctional dynamics, and consolidating the therapeutic alliance. Over the course of the sessions, a progressive increase was observed in the child’s ability to maintain regulated contact, with a reduction in impulsive behaviors during interactions.

Indicators are consistent with the following:

- Node 20 (Discordant rhythm)—difficulty of both parents and child in maintaining a stable, shared tempo during interactions.
- Node 21 (Blocked gesture)—observed in moments of suspended bodily expressiveness during emotional dysregulation.

#### 2.4.5. Phase 5

An enhancement of the child’s sensitivity and empathic capacity was observed, together with support for tolerance of setbacks and for social regulation. Compared to baseline, problem behaviors (yelling, prolonged protesting, manipulations, and verbal aggression) were significantly reduced, becoming isolated occurrences. The outcome shows greater interactive flexibility in the child, a broader range of adaptive responses, and a reduction in punitive dynamics in the parent–child relationship.

Indicators are consistent with the following:

- Node 3 (Fragmented perception of the child)—progressively overcome and replaced with a more global and affective view.
- Node 15 (Repressed impulsivity)—in the modulation of emotional and behavioral reactions.

#### 2.4.6. Phase 6

Positive outcomes emerged, along with the initiation of monitoring for the child’s transition to middle school, with maintenance of networking and continuity of support. The therapeutic contract was partially redefined to support the new developmental phase and to guide parents toward assuming a more autonomous role in managing difficulties.

Consolidation of the processes activated in the previous nodes.

### 2.5. Clinical Fragments—Case 3—Family C

#### 2.5.1. Phase 1

A case was presented of a 6-year-old child with level 3 autism, non-vocal. At the outset, limited parental adherence to treatment was observed, with frequent absences and difficulties in applying behavioral recommendations. Parents fluctuated between experiences of guilt and withdrawal, confidence and discouragement, showing an initial caution in accepting support and some hesitation toward parent training, which was gradually overcome through a motivational intervention by the clinical team.

To describe the baseline, caregivers were provided with data collection forms for 7 consecutive days, recording the frequency of topographically defined behaviors: episodes of intense screaming, physical opposition, sudden withdrawal from tasks, and relational shutdown behaviors. These behaviors were found to be very frequent and pervasive, characterizing the initial picture.

Indicators are consistent with the following:

- Node 1 (Non-integrated parental self)—in the difficulties of cohesion within the parental role.
- Node 5 (Denied relational need)—in the struggle to accept external support.
- Node 6 (Failure as identity)—in associating the child’s difficulties with self-critical parental perceptions.
- Node 13 (Who am I now?)—in the identity disorientation that may accompany the post-diagnosis phase.

#### 2.5.2. Phase 2

Work was carried out on parental role awareness and on the redefinition of family boundaries, while maintaining a realistic focus on the possibilities of the therapeutic pathway. In this phase, a therapeutic contract was formalized in which, for each identified objective, behavioral procedures (differential reinforcement, extinction), clinical–phenomenological support techniques, and expected timelines were defined. The objectives specifically concerned the reduction in oppositional behaviors and the promotion of moments of shared participation, without assuming expectations of rapid transformation.

Indicators consistent with the following:

- Node 2 (Control as defense against chaos)—in the need to maintain a stable set-up to avoid facing emotional complexity.
- Node 18 (Punitive index)—in the tendency to perceive responsibility for change as external, including in relation to the child.

#### 2.5.3. Phase 3

Emergence of deep affective resistances and motivational fragility. Building a minimal alliance to ensure continuity of the process, without forcing unsustainable openings. The intervention area remained focused on the ability to recognize the problem behaviors identified at baseline, without this leading to an observable reduction, but rather to a gradual process of parental awareness regarding their own experience.

Indicators are consistent with the following:

- Node 7 (Reactive attachment)—in the rapid shift between involvement and affective withdrawal.
- Node 17 (Refusal of need)—in the difficulty of recognizing and sharing aspects of vulnerability.

#### 2.5.4. Phase 4

Relational experiments were limited by the family system’s resistance. Targeted interventions focused on maintaining minimal contact and preventing therapeutic rupture. The monitoring forms continued to record very frequent problem behaviors, without showing substantial variations compared to baseline.

Indicators are consistent with the following:

- Node 4 (Disembodied empathy)—in the purely cognitive management of some interactions with the child.
- Node 20 (Discordant rhythm)—difficulty synchronizing with the child’s timing and modes.

#### 2.5.5. Phase 5

Process evaluation with awareness that real transformation was, at this stage, limited. Recognition of micro-movements of openness as significant results. The analysis of the collected data was used more as a tool for narrative awareness than as an indicator of change, helping parents to interpret their child’s behaviors without reducing them exclusively to deficits.

Indicators are consistent with the following:

- Node 3 (Fragmented perception of the child)—still prevalent, with few moments of unified vision.
- Node 14 (Blocked narrative of the relationship)—in the difficulty of narrating the parent–child bond in terms other than deficits or functions.

#### 2.5.6. Phase 6

Redefinition of the clinical “possible”: reflections on the quality of therapist–patient contact, acceptance of current limits, and leaving open the possibility of a more transformative work in the future. The final picture confirmed the persistence of frequent and pervasive problem behaviors, with no observable outcomes, but with greater clarity in defining the boundaries of intervention and realistic expectations.

## 3. Results

Within this section, the stratification of prompts in relation to the six phases of the protocol will be presented. The decision to present the results through the stratification of prompts across the six phases of the protocol responds to the need to show how the transformative nodes derive directly from the clinical activation of such stimuli, thereby making the process of change observable and not only its theoretical framework. The nodes are therefore not merely descriptive steps but critical transitions of change made possible by the targeted use of prompts, which guide the pathway and allow the clinician to access new forms of awareness and reorganization for improved treatment delivery.

### 3.1. Prompt Stratification

#### 3.1.1. Prompt 1

You are starting a parent training program based on the Autism Open Clinical Model (A.-O.C.M.). In your responses, specifically refer to the foundational A.-O.C.M. article and the “24 Transformative Nodes” clinical protocol. We are in Phase 1: Welcoming and Exploring the Family Context. The nodes to be addressed are as follows:

1. The non-integrated parental self

5. The denied relational need

6. Failure as identity

13. Who am I now?

These nodes define the first experiential and relational fractures observable in the initial phase of parent training. Based exclusively on these nodes, and without including elements from subsequent phases, identify the behavioral indicators that can signal, in clinical practice, the quality of the parental self. By “quality of the parental self,” we mean the parent’s ability to recognize, integrate, and embrace their dissociated parts, including the capacity to share the parental role with the other parent. For each node, identify both manifest and latent indicators. Strictly exclude nodes from other phases, even if they have conceptual similarities. There must be no semantic overlaps with previous or subsequent phases. Clearly describe the behavioral indicators to observe in relation to Phase 1: Welcoming and Exploring the Family Context.

##### Therapist’s Self-Reflection (Phenomenological Framework)

Clinical context: You are working with a parent who perceives themselves as inadequate.

Emotional activation: What did you feel in your body when they said, “I don’t know who I am anymore”?

Role in the field: Did you feel like a witness, savior, child, judge?

Threshold/paradox: If you remain in passive listening, what do you risk? If you intervene, what do you fear?

Open question: Who are you when you welcome a parent who has lost themselves?

#### 3.1.2. Prompt 2

You are a behavior analyst conducting parent training according to the A.-O.C.M. In your responses, specifically refer to the foundational A.-O.C.M. article and the “24 Transformative Nodes” clinical protocol. We are in Phase 2: Redefining Parental Identity and Expectations. The goal of this phase is to explore a possible narcissistic wound that may have occurred when parents received their child’s autism diagnosis. The focus is on emotional reactions and how these are enacted in the family context, with particular reference to the relationship with the other spouse. Consider: node 2 “Control as a defense against chaos,” node 11 “The ideal image that nullifies the real child,” node 16 “The desire for conformity,” and node 18 “The punitive index.” Strictly exclude nodes from other phases, even if they have conceptual similarities. There must be no semantic overlaps with previous or subsequent phases. Clearly describe the behavioral indicators to observe in relation to Phase 2: Redefining Parental Identity and Expectations.

##### Therapist’s Self-Reflection (Phenomenological Framework)

Clinical context: You have just concluded a session with a couple in conflict about their child’s diagnosis.

Emotional activation: What emotion lingered in you at the end of the meeting?

Role in the field: Did you position yourself as a peacemaker, expert, neutral third party?

Threshold/paradox: What happens in you if you feel the need to “correct” their expectations?

Open question: What are you protecting, inside yourself, in this field?

#### 3.1.3. Prompt 3

You are a behavior analyst conducting parent training according to the A.-O.C.M. In your responses, specifically refer to the foundational A.-O.C.M. article and the “24 Transformative Nodes” clinical protocol. We are in Phase 3: Supporting the Emotional Bond and Secure Attachment. The goal of this phase is to examine the quality of the parent–child relationship from both a manifest and latent perspective, with reference to the parent/child attachment style. Assess whether the grief of having an autistic child has been processed. The emphasis is on the quality of contact. Also explore the possible existence of triangulation dynamics. Refer to the following nodes: 7 “Reactive attachment”; 10 “The child as a figure of unprocessed grief”; 12 “The child as a space interposed between parents”; 17 “The refusal of need.” For each node, identify both manifest and latent indicators. Strictly exclude nodes from other phases, even if they have conceptual similarities. There must be no semantic overlaps with previous or subsequent phases. Clearly describe the behavioral indicators to observe in relation to Phase 3: Supporting the Emotional Bond and Secure Attachment.

##### Therapist’s Self-Reflection (Phenomenological Framework)

Clinical context: During today’s session, the parent’s responses to the child varied between engagement and withdrawal.

Emotional activation: What did you feel in your body in front of this oscillation?

Role in the field: At that moment, who were you for them? An observer? A missing father?

Threshold/paradox: If you try to harmonize the bond, what do you fear might emerge?

Open question: Which part of your relational history are you brushing against in this passage?

#### 3.1.4. Prompt 4

You are a behavior analyst conducting parent training according to the A.-O.C.M. In your responses, specifically refer to the foundational A.-O.C.M. article and the “24 Transformative Nodes” clinical protocol. We are in Phase 4: Integration of the Body, Empathy, and Embodied Communication. The goal of this phase is to explore the level of cognitive empathy and affective empathy in parents. These aspects are distinct and not necessarily coinciding. The focus is on the body language that expresses emotions not always consciously recognized. Emphasis is placed on how each caregiver’s sensory systems function in interaction with the child. Refer to the following nodes: 4 “Disembodied empathy”; 8 “Disconnected parental body”; 19 “The body as an alien place”; 20 “Out-of-sync rhythm”; 21 “Blocked gesture”; 22 “Traumatic touch”; 23 “The dissociated embodiment of the child”; 24 “Interrupted sensory dialogue.” For each node, identify both manifest and latent indicators. Strictly exclude nodes from other phases, even if they have conceptual similarities. There must be no semantic overlaps with previous or subsequent phases. Clearly describe the behavioral indicators to observe in relation to Phase 4: Integration of the Body, Empathy, and Embodied Communication.

##### Therapist’s Self-Reflection (Phenomenological Framework)

Clinical context: You are observing a father who never touches his child.

Emotional activation: What bodily reaction did you have to their sensory distance?

Role in the field: Did you feel part of the scene, or an observer outside the body?

Threshold/paradox: If you tune in too much to the child’s body, what do you risk in yourself?

Open question: How did your body speak to you during the interaction?

#### 3.1.5. Prompt 5

You are a behavior analyst conducting parent training according to the A.-O.C.M. In your responses, specifically refer to the foundational A.-O.C.M. article and the “24 Transformative Nodes” clinical protocol. We are in Phase 5: Symbolic Reorganization and Shared Narrative. This phase explores in detail how the child is perceived through the verbal channel expressed in the therapeutic setting and through the examination of the unspoken. The latter highlights the pre-reflective quality of the mind as an embodied product of the body. The body functions as a resonance chamber. This phase centers on repressed emotions. Here, the reading is through the embodied mind paradigm without neglecting the other principles of the A.-O.C.M. Refer to the following nodes: 3 “The fragmented perception of the child”; 9 “The block of symbolic generalization”; 14 “The blocked narrative of the relationship”; 15 “Repressed impulsivity.” For each node, identify both manifest and latent indicators. Strictly exclude nodes from other phases, even if they have conceptual similarities. There must be no semantic overlaps with previous or subsequent phases. Clearly describe the behavioral indicators to observe in relation to Phase 5: Symbolic Reorganization and Shared Narrative.

##### Therapist’s Self-Reflection (Phenomenological Framework)

Clinical context: You have listened to a parent describing the child only as a set of symptoms.

Emotional activation: Which word touched you more than expected?

Role in the field: Did you feel like a clinician, spectator, substitute narrator?

Threshold/paradox: If you try to return a more unified vision, what do you fear might break?

Open question: What is your body really telling while listening to those words?

#### 3.1.6. Prompt 6

You are a behavior analyst conducting parent training according to the A.-O.C.M. In your responses, specifically refer to the foundational A.-O.C.M. article and the “24 Transformative Nodes” clinical protocol. We are in Phase 6: Closure and Opening to the Future. This phase embraces and synthesizes the processes activated in the previous phases, preparing for closure and the next evolutionary step. The clinician drafts a prompt containing the following: (1) concisely report the objectives present in the therapy contract; (2) concisely describe the baseline: the initial clinical picture; (3) concisely describe the objectives achieved; (4) instruct the device with the following command: “give me honest and rigorous feedback on this outcome, remaining within the A.-O.C.M. framework.”

##### Therapist’s Self-Reflection (Phenomenological Framework)

Clinical context: You have just concluded a therapeutic path and are reflecting on the outcomes.

Emotional activation: What emotion inhabited your body during the closure?

Role in the field: Did you remain a therapist, become a witness, or feel emotionally involved?

Threshold/paradox: If you recognize the transformation that has occurred, what do you feel belongs to you?

Open question: What have you discovered about yourself that you had never seen in therapy [17,18]?

##### Operational Implications for Protocol Implementation

As highlighted in Appendix A, it is crucial not to modify the architecture of the prompts in their original formulation. The development of these tasks was built on a technological–scientific literature base that defines specific clinical prompt design principles, which determine the quality of the outputs. The content of the prompts, supported by extensive and updated scientific data, draws upon the authors’ long-standing clinical experience with autism and is based on the six-phase clinical protocol with the corresponding transformative nodes included in each phase.

The analysis of clinical fragments from Case 1 was particularly useful for refining these prompts. Subsequently, through the implementation of parent training with the spouses in Cases 2 and 3, the process of validating the stratified prompt protocol began.

This proposal fits within a research architecture based on a clearly defined hypothesis and a rigorously structured methodological approach, representing a preliminary phase toward designing a controlled experimental study. It is also essential that the user’s device permanently store specific commands (Appendix B), files, and notes (Appendix C).

Compliance with the operational guidelines described above is a necessary condition for implementing the protocol.

The following clinical cases concretely illustrate how the phases and nodes manifest in practice, providing direct observational examples.

## 4. Discussion

The integration of a trained artificial intelligence device, particularly through the use of the “Therapeutic Self” prompt, represented a significant clinical contribution to the evolution of the cases presented, maintaining continuity with the theoretical framework outlined earlier. The dialogical structure generated by this tool enabled the activation of a dynamic process of intervision, in which case observation was interwoven with a reflective and embodied analysis of the clinician’s position within the relational field.

In Case 1, the use of the device primarily supported the clarification of the parental self and the redefinition of family boundaries. The stratified prompts played a crucial role: through their graduated sequence, parents were guided to more precisely distinguish observable problem behaviors from those perceived as such on an emotional level. This differentiation reduced the initial confusion, providing a shared framework within which to redefine the reported difficulties.

In particular, the prompts operated as orienting stimuli, capable of supporting parents in a process of progressively objectifying their experiences. The use of data collection forms, integrated with the feedback emerging from the work on the “Therapeutic Self,” made it possible to transform defensive and projective dynamics into observable and shareable indicators. In this way, the process fostered greater symbolic integration and supported a progressive reduction in problem behaviors, both in their frequency and in their perceived intensity.

In Case 2, the prompts developed according to the modular architecture of the A.-O.C.M. offered a refined interpretive framework of both manifest and latent indicators, promoting a more stratified and rigorous clinical observation. Access to such an articulated perspective would have been more challenging without the aid of a tool capable of functioning as a “third eye” within the process. This function made it possible to distance the analysis from self-reflection alone, reducing the risk of selective blind spots and offering an external yet integrated perspective, while maintaining phenomenological grounding in experience.

In Case 3, the interactive reflection on the Therapeutic Self made it possible to break out of a relational impasse: as a team, we found ourselves in a closed dimension, characterized by bodily sensations of suffocation and lack of breath. At that moment, there was no authentic contact with the couple. Within this framework, the absence of mutual recognition prevented any real transformative movement. The work with the device brought this fracture to light, revealing that without contact, there can be no change, and that such contact requires genuine awareness generated by authentic insights rather than unprocessed introjections. This realization opened a space of ethical questioning about the boundary between working with resistances and falling into therapeutic overinsistence, with possible iatrogenic outcomes.

AI did not, therefore, replace clinical work but functioned as a catalyst: it amplified the therapist’s capacity to remain with the experience and to perceive the subtle movements of the field, integrating the clinical practice already described with targeted stimuli, coherent feedback, and phenomenological questioning. This process enhanced the quality of clinical awareness, supporting transformative work that involved not only the caregivers but also the bodily and ethical positioning of the therapist within the relationship, in line with the founding principles of the A.-O.C.M.

We are aware that, although the clinical outcomes observed were consistent with expectations, further clinical implementation and a larger number of studies are needed to establish the extent to which the presented protocol can be replicated, generalized, and capable of accounting for the multiplicity of diagnostic and process variables. In this sense, the adopted methodology is eclectic yet scientifically oriented. However, for an eclectic approach to be fully defined as evidence-based, it requires repeated testing and empirical validation conducted on larger clinical samples.

The presented protocol, understood as a preparatory phase, outlines an integrated clinical model whose validity can only be fully attested through structured feasibility and effectiveness studies, carried out on larger samples and across diverse contexts. Our aim is to overcome the risk of remaining confined to case series or supervisory descriptions, transforming the clinical framework into an experimental program that is verifiable and replicable.

### 4.1. Strengths and Limitations

#### 4.1.1. Strengths of the Proposed Protocol

(a) Architecture and traceability: the articulation into six phases and 24 nodes with stratified prompts generates an operational “audit trail” (which node was activated, with which prompt, in response to which indicators) that supports implementation fidelity and replicability; these aspects will be evaluated using established fidelity frameworks (checklists, supervision, inter-rater evaluations).
(b) Alignment with evidence on parent-mediated interventions: the literature shows that training focused on parental responsiveness and communication can produce sustained improvements in communicative outcomes and, in some cases, in symptom severity at multi-year follow-up. The A.-O.C.M. leverages the same principles of engagement, co-regulation, and generalization in natural contexts.
(c) Ecology and scalability: the model prioritizes observable and routine-based objectives, compatible with pragmatic designs and with metrics of adoption and maintenance, thereby facilitating implementation across heterogeneous services.
(d) Originality and theoretical–methodological grounding: the international literature on the integration of psychology and artificial intelligence is rapidly developing but still at an early stage. Some studies have explored forms of integration between parent training and AI, but not with specific reference to autism and, above all, without the formalization of a structured clinical protocol anchored in a theoretical–methodological framework [7,8]. To date, the tools described in the literature have mainly been conceived as technological supports at the margins of clinical work, not as devices integrated into a scientifically grounded model. In this sense, the originality of the presented protocol lies in having translated an epistemological and methodological framework (A.-O.C.M.) into an operational tool articulated into phases and transformative nodes, establishing the basis for its traceability and replicability.

An additional strength lies in its structural and methodological flexibility, which distinguishes it from standardized and rigid approaches. Although articulated and complex, the designed parent training was conceived as a dynamic and adaptable tool, capable of modulating its application according to the specificities of each family unit.

Flexibility manifests itself on multiple levels:

Structural: the six phases and twenty-four transformative nodes are not rigid sequential constraints but constitute a clinical map that can be traversed along different trajectories, depending on the urgencies and resources of the context.

Methodological: the integrated techniques (ABA, phenomenology, Gestalt, and the embodied mind paradigm) make it possible to modulate the focus of the intervention, prioritizing behavioral, relational, bodily, or narrative dimensions depending on the case.

Evolutionary: the framework entails a constant process of monitoring and revision, allowing objectives and strategies to be readapted according to observed changes in the child, the parents, and the family system.

#### 4.1.2. Limitations

(a) Clinical heterogeneity and risk of therapist drift: the wide variability of family needs may dilute the effect; manuals, calibration sessions, and independent fidelity monitoring are planned to contain undesired variance.
(b) Measurement of processes: indices of parent–child interaction require sophisticated methodologies and coding systems; the plan includes evaluator training and blinded observation to reduce performance and detection bias.
(c) Specialized training requirements: the model, addressed specifically to scholars in the field, necessarily requires training in the use of prompts and supervision. Moreover, in order to correctly understand and implement the tool, phenomenological training and advanced competencies in Applied Behavior Analysis are needed. In the absence of these prerequisites, the tool remains difficult to access.

### 4.2. Research Perspectives

In light of the strengths and limitations identified, the next step will be the manualization of the A.-O.C.M. protocol, translating the six phases and twenty-four nodes into operational procedures, fidelity checklists, and observational grids. This process, conceived as a methodological deepening, will ensure a consistency of application across different therapists and provide useful tools for clinical training and supervision.

A second level of development concerns the implementation of feasibility studies, designed as pilot phases on small samples (15–30 families), aimed at evaluating the practicality of the protocol, the degree of adherence and acceptability by caregivers, as well as the fidelity of implementation by practitioners. These studies will allow the collection of preliminary data, the identification of potential organizational barriers, and the monitoring of the initial effects of the transformative nodes within the family context [19].

Only subsequently will it be possible to conduct effectiveness studies, using designs differentiated according to available resources: pragmatic randomized trials when sample sizes and contexts permit, or multiple-baseline designs on single cases and families when working in small clinical settings. In both cases, reporting will need to comply with CONSORT standards [20] and their extension for pragmatic trials, in order to ensure transparency, comparability, and methodological rigor [17].

## 5. Conclusions

The A.-O.C.M. and the protocol described here represent an evolution of parent training: from a prescriptive technique to an embodied, adaptive, and documentable process. “Without the body there is no clinic, without method there is no science” [18].

Below is Table 1, which provides a summary of the appendices for the implementation of the A.O.C.M. protocol.

## Figures and Tables

**Table 1:** Synthesis of the appendices for the implementation of the A.-O.C.M. protocol.

Level of Implementation	Concise Description	Function in Implementation
Principles of Integrity and Consistency (Appendix A)	The A.-O.C.M. protocol requires that each phase and transformative node be applied rigorously: exclusion of unrelated nodes, accuracy in naming, necessary redundancy in prompts, and strict adherence to the phenomenological and behavioral structure.	Defines the methodological framework and guarantees that prompts respect the original clinical structure without alterations.
Permanent Operational Commands (Appendix B)	Permanent commands ensure uniformity: updated literature (2015–2025, indexed sources), mandatory references (Cooper, Skinner, Husserl, Merleau-Ponty, Francesetti), and integrity checks prior to document delivery.	Establishes operational rules and epistemological constraints that make the model standardizable and replicable.
Archive and Structural References (Appendix C)	The permanent archive stores foundational articles (Morfini 2023 [9]), protocol extracts, and clinical notes on the 24 nodes across the 6 phases. It provides a stable basis to ensure replicability, epistemological coherence, and shared access between therapist and AI.	Builds the clinical and theoretical memory that integrates ABA, phenomenology, and embodied mind, ensuring continuity and traceability of the protocol.

## Data Availability

The data supporting the findings of this study are available from the corresponding author upon reasonable request (psiconapoli@gmail.com). Due to privacy and ethical considerations, the data are not publicly accessible.

## Appendix A. Principles of Integrity and Consistency in the Execution of Clinical Prompts

The prompts indicated in the subsection of the Section 2 must be followed literally, as their architecture adheres to technological principles of clinical prompt design. The development of these prompts has followed a control checklist, outlined below:

- The files have been correctly uploaded, each bearing a clear and recognizable name for the user.
- The transformative nodes linked to each phase must be explicitly stated using their correct designation.
- It is necessary to specify the exclusion of nodes that belong to other phases, even if semantic overlaps exist.
- A certain degree of redundancy in the production of prompts is considered necessary to significantly reduce the risk of generating inaccurate outputs.

## Appendix B. Operational Commands to Be Stored in the Device’s Permanent Memory

### Appendix B.1. Command 1

When referring to the scientific literature, the following apply:

- Use articles published in the last 10 years (2015–2025).
- Prefer sources from internationally indexed journals (Scopus/Web of Science), including DOI and essential bibliometric data (IF/SJR where useful).

### Appendix B.2. Command 2

When referring to ABA programs and treatment plans, the following apply:

- Always use the following as key reference texts:
  1. Cooper, Heron, Heward—Applied Behavior Analysis (most recent edition).
  2. B.F. Skinner—seminal works on verbal behavior, operant conditioning, and learning principles.
- Integrate with scientific articles from the last 10 years, but ensure main theoretical concepts and definitions remain anchored to Cooper.
- The function of behavior aimed at gaining attention must be distinguished from the function of gaining control of the relationship. These are distinct functions in the scientific literature and these should not be conflated.

### Appendix B.3. Command 3

When referring to the phenomenological and Gestalt literature, use the following as key reference texts:

Phenomenology—classic works among the most authoritative:

- Edmund Husserl—Ideas for a Pure Phenomenology; Cartesian Meditations.
- Martin Heidegger—Being and Time.
- Maurice Merleau-Ponty—Phenomenology of Perception.

Gestalt Therapy:

- Fritz Perls, Ralph Hefferline, Paul Goodman—Gestalt Therapy: Excitement and Growth in the Human Personality.
- Gianni Francesetti—works on psychopathology and phenomenology in Gestalt Therapy (Psychopathology of the Situation; Diagnosis in Gestalt Psychotherapy), with a focus on the situational perspective and co-construction of experience.

Integration will always be carried out by anchoring phenomenological and Gestalt concepts to the active principles of the A-O.C.M.

### Appendix B.4. Command 4

Integrity check before Word file delivery:

(1) Content integrity
  - No cuts or additions not requested.
  - Order and organization identical to the original delivery, unless explicit instructions state otherwise.
  - Tables complete (no summaries), numbers/lists preserved.
(2) Compliance with prompt requirements
  - Literature: only 2015–2025, indexed journals, DOI (Command 1).
  - ABA: anchored to Cooper, Heron, Heward, and Skinner for definitions and programs (Command 2).
  - Gestalt/phenomenology: A-O.C.M. framework + classics (Husserl, Heidegger, Merleau-Ponty, Sartre, Scheler) + Francesetti when relevant (Command 3).

### Appendix B.5. Command 5

If you encounter difficulties in executing a requested operation correctly, stop and ask how to proceed.

## Appendix C. Archive of Files and Notes for Permanent Device Memory

- Morfini, F. Autism Open Clinical Model (A.O.C.M.): Structure of an Integrated Clinical Model for the Assessment and Treatment of Autism. Phenomena Journal—International Journal of Psychopathology, Neuroscience and Psychotherapy 2023, 5(2), 1–14. https://doi.org/10.32069/PJ.2021.2.184 (foundational article of the model).
- Extract from this article: Protocol Phases (subsection of the Section 2).
- Extract from this article: Protocol Phases and Transformative Nodes (subsection of the Section 2).

Store in the device’s permanent memory the following note regarding recent developments of the A.-O.C.M. This note is not part of a published work but is written privately by the authors, and it will be included in a forthcoming scientific publication.

The Autism Open Clinical Model (A-O.C.M.) is an integrated clinical model designed to address the complexity of autism spectrum disorders through a systemic, dynamic, and multidimensional approach. Since its inception, it has aimed to bridge the gap between scientific research and everyday clinical practice, still too anchored to rigid, poorly personalized protocols, placing the autistic person and their family at the center of the process.

In its most recent developments, the A-O.C.M. has embraced the paradigm of the embodied mind as a further theoretical and operational horizon, capable of adding coherence and depth to its structure. The embodied mind—understood as the inseparable intertwining of body, emotions, perceptions, and relationships—is not merely a theoretical reference but becomes a constitutive part of clinical work. In this perspective, every experience, behavior, or opportunity for learning is rooted in a living body and in a relational context, and therapy is based on listening to and activating these dimensions.

Gestalt Therapy, already a guiding principle of the model, thus finds an expanded framework: the centrality of the organismic field, contact, and the co-construction of meaning integrates with the phenomenological vision of the embodied mind. Clinical work becomes a meeting between two bodily and aware presences, where attention is not limited to the function of behavior but extends to the way the patient’s body inhabits space, time, and relationships.

Integration with the embodied mind further strengthens the open nature of the A-O.C.M.:

- Epistemological openness, welcoming verifiable scientific and clinical contributions;
- Openness to dialogue between models, where ABA, CBT, and Gestalt tools are enriched with practices oriented to bodily and sensory experience;
- Relational openness, recognizing evolutionary pairing as the construction of an affective and regulatory field;
- Openness to the evolutionary uniqueness of each individual, understood not only in cognitive terms but also bodily and perceptual.

The result is an embodied and dialogical model, in which the rigor of functional analysis coexists with the depth of phenomenological experience, and where change is not imposed from the outside but arises from the resonance between the body, mind, and relationships. This evolution consolidates the A-O.C.M. as a generative clinical structure, capable of adapting and growing, while keeping its roots firmly grounded in the real-life experiences of individuals.
